# Disinfection of Bacteria in Aerosols by Applying High Voltage to Stranded Wire Electrodes

**DOI:** 10.3390/microorganisms12020418

**Published:** 2024-02-19

**Authors:** Takahisa Ueno, Konosuke Takada, Shohei Zaizen, Takashi Sakugawa, Junko Ninomiya, Takashi Furukawa

**Affiliations:** 1 Department of Electrical and Electronic Engineering, National Institute of Technology, Oita College, 1666 Maki, Oita-shi 870-0152, Japan; 2 Electrical, Electronic and Information Engineering Major, Advanced Course, National Institute of Technology, Oita College, 1666 Maki, Oita-shi 870-0152, Japan; 3 Institute of Industrial Nanomaterials, Kumamoto University, Kurokami 2-39-1, Chuo-Ku, Kumamoto-shi 860-8555, Japan; sakugawa@cs.kumamoto-u.ac.jp; 4 General Education, National Institute of Technology, Oita College, 1666 Maki, Oita-shi 870-0152, Japan; j-ninomiya@oita-ct.ac.jp; 5 Department of Health Science, School of Allied Health Sciences, Kitasato University, A1-505, 1-15-1 Kitasato, Minami-Ku, Sagamihara-shi 252-0373, Japan; tfuruka@kitasato-u.ac.jp

**Keywords:** impulse voltage, pulsed electric field (PEF), disinfection of bacteria in aerosol, stranded wire electrode

## Abstract

The inactivation of airborne pathogenic microorganisms is crucial to attenuate the dissemination of infectious diseases induced by airborne pathogens. Conventional air disinfection methodologies, such as ultraviolet (UV) irradiation and ozone treatment, have demonstrated limited efficacy. Consequently, we investigated the potential of employing pulsed voltages to effectively eradicate bacteria within aerosols. Our inquiry revealed that the bacterial disinfection rate increased proportionally with elevated applied voltage and frequency. For instance, when a pulsed voltage of 20 kV and a frequency of 500 Hz were applied, a substantial disinfection rate exceeding 6.0 logarithmic units was attained. Furthermore, with the utilization of the stranded wire anodes, the disinfection intensity could be augmented by up to 2.0 logarithmic units compared with the solid wire configuration. Through the utilization of a stranded wire electrode model, we scrutinized the electric field encompassing the electrode, revealing a non-uniform electric field with the stranded wire electrode. This observation indicated an amplified bacterial disinfection effect, aligning with our experimental outcomes. These findings significantly enhance our comprehension of efficacious approaches to electrically disinfecting airborne bacteria.

## 1. Introduction

The novel coronavirus precipitated a global pandemic, wielding a profound impact [1]. Despite indications of mitigation, the apprehension surrounding the prospect of a new pandemic, extending beyond coronaviruses, remains unabated. Modes of transmission for these infections share commonalities with other pathogens such as the measles virus and Mycobacterium tuberculosis [2,3,4]. Specifically, these transmission mechanisms encompass contact transmission and spaceborne transmission. Spaceborne infections encompass droplet infections for particles with droplet nuclei exceeding 5 μm and airborne infections for those with nuclei smaller than 5 μm [5]. As a result, the inactivation of aerial pathogenic microorganisms has assumed paramount significance, catalyzing the quest for diverse infection control measures [6]. Conventional means of disinfection, encompassing aerial chemical spraying and atomized disinfectant suspensions, target both airborne and surface-bound bacteria [7,8,9,10]. Additionally, ultraviolet (UV) and ozone sterilization have been pivotal in enclosed spaces [11,12,13,14,15]. The bactericidal efficiency of UV radiation, particularly in the 260 nm wavelength range, is attributed to its resonance with the absorption spectrum of DNA, leading to hydration, dimer formation, and decomposition, thus eliminating fungi [16]. Given that the light absorption spectrum of DNA closely approximates that of the bactericidal wavelength region, it is postulated that ultraviolet irradiation triggers hydration, dimer formation, and decomposition, culminating in the eradication of fungi [17,18]. Ultraviolet irradiation is distinguished for its efficiency in sterilizing various bacterial strains and is particularly noteworthy for its non-residual nature, as it does not involve chemical agents. This method generates minimal by-products, allows sterilization at ambient temperatures, induces minimal alterations to irradiated materials, and mitigates the emergence of resistant bacterial strains.

Ozone sterilization operates by disintegrating bacterial cell walls and oxidatively decomposing cell surface components. Vulnerable cell components encompass lipoproteins and lipopolysaccharides within cell walls, as well as phospholipids, lipoproteins, lipopolysaccharides in the cell membrane, enzyme proteins, coenzyme constituents, and nucleic acids within the cytosolic compartment and cytosol [11]. Ozone sterilization exhibits recognized effectiveness against bacteria, yeast, mold, and viruses. It is utilized in the sterilization of food and food constituents, the purification of air in food manufacturing facilities and storage units, and the meticulous cleansing of organic matters.

Several studies have explored ultraviolet and ozone sterilization [11,19,20,21,22,23,24], and their practicability continues to evolve. However, ultraviolet rays may not deliver sufficient sterilization over a broad area due to scattering and aerosol absorption [25]. Additionally, the efficacy of ultraviolet rays can be impeded by turbidity and obstacles, restricting their reach. Ozone, with a 1% (wt) concentration in dry air, has a half-life of approximately 16 h, decreasing with higher humidity [11]. The impact of ozone on living organisms is profound, presenting as toxicity, skin/eye irritation, carcinogenicity, and mutagenicity to DNA and bacteria. Environmental ozone concentrations typically range from 0.03–0.06 ppm in coastal regions, 0.005–0.02 ppm at ground level, to 0.05–0.10 ppm in forested areas. At concentrations of 1–2 ppm, fatigue and respiratory function changes ensue, and at 50 ppm or higher, life-threatening effects may occur [11,26]. Henceforth, following contemplation of the repercussions on the human physiological system and the imperative for comprehensive sterilization, ozone and ultraviolet irradiation were proven unsuitable for the sterilization of environments frequented by human occupants. 

In this study, we introduce a novel disinfection approach: the utilization of electric fields for disinfecting bacteria within enclosed spaces. In recent years, a burgeoning field known as bioelectronics has emerged. Bioelectronics encompasses diverse areas, including the manipulation of living organisms using electricity and its combination with ecologically relevant substances [21,27,28,29,30,31]. Of particular note is the technique of microbial sterilization and manipulation using pulsed electric fields, known as Pulsed Electric Field (PEF), which is gaining attention due to its non-thermal sterilization capabilities without reliance on chemicals.

Numerous PEF studies have been conducted, including experiments by Sale and Hamilton [32], especially concerning liquid sterilization. Sato et al. (2019) affirmed that the application time of a DC electric field did not impact the viability of *Escherichia coli* (*E. coli*) in a sodium chloride suspension [33]. Ishida et al. (2004) devised a spiral-shaped electrode treatment layer for pulse voltage sterilization in liquids, examining factors like the capacitance of the pulse generator power supply, suspension conductivity, treatment layer volume, and electrode material. Their findings underscored the significant influence of applied voltage, capacitor capacitance, and suspension conductivity on viability, while the treatment layer volume and the electrode connection method had no effect on viability [34]. Furthermore, Uemura et al. (2016) and Nakamura et al. (2021) achieved a 4.0 logarithmic reduction through the pulsed electric field pasteurization of orange juice and milk, respectively, surpassing the efficacy of high-temperature treatments [35,36]. 

Space sterilization via electric fields employs high-voltage electrical discharges to eliminate microorganisms within an airspace, such as a hospital room or laboratory. Plasma, generated by the electric field, produces active species like ozone, hydroxyl radicals, and reactive oxygen species, which interact with microorganisms and result in their demise. Lee et al. (2006) explored the utilization of electric fields for space sterilization. They irradiated *E. coli* and *Staphylococcus aureus* (*S. aureus*) with helium/oxygen plasma generated by AC power at a frequency of 10 kHz and 6 kV, achieving over 90% sterilization within just 18 s [37]. Another study by Nakamura et al. (2009) utilized discharge plasma to generate ions and hydroxyl radicals, effectively removing 99% of airborne bacteria within a 40 m^3^ space in 38 min [38]. Zhang et al. (2022) investigated aerosol bacteria sterilization using dielectric barrier discharges with a pulsed electric field at a frequency of 1 kHz. Their results indicated that pulse-driven dielectric barrier discharges achieved an elimination of over 90% of *E. coli* and *Staphylococcus albus* (*S. albus*) within the space, outperforming AC voltage-driven dielectric barrier discharges at the same power level [39]. Overall, spatial sterilization using electric fields presents a promising and effective method for eliminating microorganisms within confined areas.

Nevertheless, further research is vital to ascertain the optimal parameters of this technology and evaluate its efficacy across various environments and microorganisms. The primary mechanism underlying voltage-applied sterilization is the disruption of cell membranes by the electric field. Enhancing the sterilization effect may involve increasing the electric field strength or concentrating the field on the cell membrane. Studies by Hayamizu et al. (1989) and Ramaswamy et al. (2019) explored electrode shapes and materials, with Hayamizu et al. investigating tissue destruction around electrodes using plate and coaxial needle electrodes, and Ramaswamy et al. comparing titanium and stainless-steel electrodes, revealing that, at an equivalent field strength of 24 kV/cm, the titanium electrode displayed superior effectiveness in killing two types of microorganisms at lower temperatures [40,41]. Kitajima et al. (2007) demonstrated that sterilization rates depend on factors such as fiber electrode wire gap length, bacterial suspension conductivity, electrode surface area, and flow velocity [42]. Modifying the electrode structure to induce electric field concentration is considered an effective strategy.

The optimal pulse waveform for efficient sterilization through pulsed electric fields has been discussed by Schoenbach et al. (2000), Miyazaki et al. (2020), and Bai-Lin et al. (1994). They examined parameters such as pulse shape, amplitude, and duration, including exponential decay waveform, oscillatory decay waveform, and square wave. Bai-Lin et al. (1994) determined that the bipolar square wave pulse voltage is the most efficient [43,44,45].

As aforementioned, pulsed electric field sterilization methodologies have been postulated to attain sterilization of elevated intensity. Nevertheless, these methodologies predominantly address bacteria in liquid media, with scant scholarly exploration dedicated to sterilization in gaseous phases. While certain studies have been documented regarding the decontamination of spaces through the employment of electrostatic fields, the emphasis has been placed on diminishing the prevalence of suspended bacteria and augmenting dust-collection efficiency [46,47].

In the current inquiry, our attention is oriented towards bacteria suspended within a spatial milieu, wherein we advocate an approach employing pulsed electric fields to amalgamate aerosols housing these microorganisms and subsequently execute sterilization. Additionally, we conducted a quantitative assessment of this methodology. Moreover, we elucidate that the electric field’s intensity can be heightened through alterations in the electrode structure, a phenomenon we validate through simulation. A comprehensive account of these findings is delineated herein.

## 2. Materials and Methods

### 2.1. Experimental Setup

**Figure 1:**
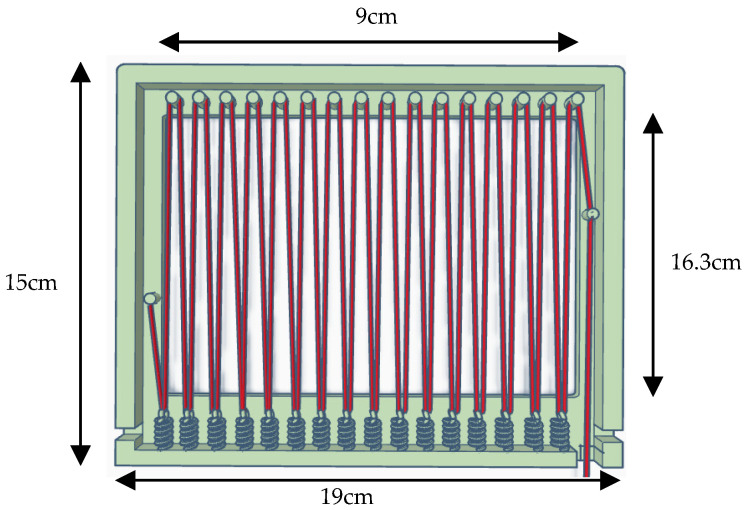
Anode Model Diagram. The red wire represents the stainless-steel wire indicating the anode. Solid wire and 2, 3, and 4 stranded wires were used for the wire electrodes.

The cathode utilized in our experimental investigations comprised a stainless-steel mesh electrode with a mesh count of 16, measuring 9 cm in length and 16.3 cm in width. The anode, on the other hand, consisted of a base measuring 15 cm in length and 19 cm in width, with a frame of 9 cm in length and 16.3 cm in width. Stainless-steel wires underwent 32 folds at intervals of 10 cm, resulting in an aggregate length of approximately 319 cm (Figure 1). For diverse experimental conditions, we employed both individual stainless-steel wires and assemblies of two to four strands of interwoven wires as the wire electrodes. The anode and cathode were systematically aligned in parallel, maintaining a separation distance of 1 cm within the experimental apparatus.

**Figure 2:**
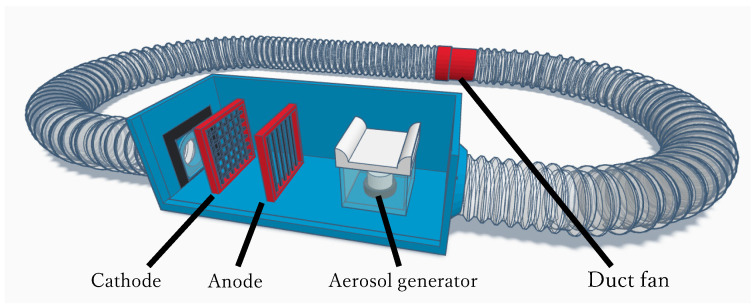
Schematic Diagram of the Experimental Apparatus. Electrodes and an aerosol generator are installed within the blue box, while a circulating reactor and duct fan are attached to the outer frame of the apparatus.

As delineated in Figure 2, a recirculating reactor was affixed to the outer periphery of the apparatus, housing a duct fan (SAILFLO IPX2, Xiamen Usail Industry And Trade, Xiamen, China) with a diameter of 10 cm. The airflow velocity within the apparatus was meticulously regulated to approximately 1.27 m/s using this duct fan. To introduce aerosols into the experimental setup, an ultrasonic aerosol generator (AGPTEK HS0056, Shenzhen Mambate Industry Development, Shenzhen, China) was submerged in a water reservoir filled with a bacterial suspension. This ultrasonic aerosol generator employed ultrasonic atomization to transmute the bacterial suspension into an aerosol, thereby saturating the experimental apparatus with the aerosol. The ambient temperature and the temperature within the containment vessel were established at 15 °C. Aerosols were generated until condensation ensued, achieving maximal humidity, as indicated by a hygrometer (DT-3321, MK scientific, Kanagawa, Japan), reaching 100%.

### 2.2. Experimental Procedure

*E. coli* ATCC11229 was used as a model bacterium in the disinfection experiment. *E. coli*, during the advanced phase of its late logarithmic growth phase characterized by heightened metabolic activity, was employed in disinfection experiments wherein impulse voltage was applied. The optimal incubation period of 24 h was ascertained based on the *E. coli* growth curve. The strain was introduced into *Luria-Bertani* broth (BD Difco, Franklin Lakes, NJ, USA) and incubated for 24 h at 35 °C ± 1.0 °C in a rotary shaker (120 rpm). Subsequently, 5 mL of the precultured *E. coli* strain underwent centrifugation at 4000× *g* for 5 min, and the supernatant was decanted. The bacterial pellet underwent two washes with 10 mL of sterile ultrapure water. The pellet was then reconstituted in 40 mL of sterile ultrapure water. The absorbance of the bacterial suspension was gauged at 600 nm (OD600) using a spectrophotometer (AE-350, Erma, Saitama, Japan). Finally, the *E. coli*-containing suspension was adjusted to approximately 3.0 × 10^6^ CFU/mL through dilution based on the calibration curve (OD600 absorbance vs. *E. coli* count). This procedure was iterated at bacterial concentrations of 10^8^ and 10^3^ CFU/mL to scrutinize the impact of the initial bacterial count on the disinfection rate. The suspension was stored at 25 °C ± 1.0 °C until application in the experimental phase. The culturable *E. coli* in each sample underwent enumeration using the spread plate method and LB Agar (71752, Sigma-Aldrich–MERCK, St. Louis, MO, USA) plates. Samples anticipated to possess elevated bacterial concentrations were diluted with sterile phosphate-buffered saline; 100 µL of the sample or its prepared dilutions were inoculated on agar plates in triplicate and incubated at 35.0 °C ± 1.0 °C for 20–24 h. This process was iterated five times for each condition. The disinfection rate was quantified as the logarithm of the ratio of the number of bacteria (*C*_0_) pre-application to the number of bacteria (*C*) post-application, as elucidated in Equation (1).

(1) $${log}_{10}(\frac{C_{0}}{C})$$

### 2.3. Comparison of Disinfection Rates at under Various Voltage Waveforms

In this experimental endeavor, a solitary strand of stainless-steel measuring 0.16 mm in diameter served as the anode. Varied voltage waveforms were imposed to assess the disinfection efficacy. Specifically, direct current (DC) voltages of 15 kV and 20 kV, alternating current (AC) voltages of 15 kV and 20 kV at a frequency of 60 Hz, and pulsed voltages of 15 kV and 20 kV at a frequency of 100 Hz were applied. The voltage parameters utilized in these experiments represented the maximum threshold, ensuring the absence of discharge between the electrodes, and the minimum threshold, facilitating the power supply to furnish a sustained output. The application duration spanned 20 min, representing the minimal time within which the bacterial suspension could be procured from the anode. The disinfection efficiencies resulting from the application of these diverse voltage waveforms were scrutinized. Furthermore, the disinfection rates achieved with the 15 kV and 20 kV pulsed voltages were juxtaposed across the frequencies of 1 Hz, 10 Hz, 100 Hz, 500 Hz, and 1 kHz.

### 2.4. Comparative Analysis of Disinfection Rates with Single and Stranded Anode Wires

In this portion of our investigation, the anode was configured in two distinct ways. It consisted of either a solitary wire composed of 0.16 mm stainless-steel or an arrangement comprising two, three, or four strands of 0.16 mm stainless-steel wire. The external frame, where these wires were securely mounted, was hollowed out to dimensions measuring 9 cm in length and 16.3 cm in width. The stainless-steel wires, fulfilling their role as the anode, were positioned atop this framework, with foldbacks occurring at intervals of 12 cm. The inter wire spacing was precisely maintained at 10 mm. To assess the impact of this configuration, pulsed voltages of 15 kV and 20 kV were applied at a frequency of 10 Hz for a duration of 20 min. Subsequently, the disinfection rate was computed according to the stipulated experimental procedure. Moreover, the temperature of the bacterial diluent solution was assessed both antecedent to and subsequent to the application of voltage, with the aim of examining the influence of temperature on the disinfection process.

### 2.5. Electric Field Analysis of Single and Stranded Wire Configurations

In our quest to comprehend the distribution of the electric field within an electrode by employing a twisted linear electrode configuration, simulations were conducted. The electrostatic field simulator employed was ANSYS Maxwell (Ansys, Cannonsburg, PA, USA), specifically utilized for computing the electrostatic field resultant from potential difference. In the context of Maxwell 3D, the fundamental unit of finite elements takes the form of a tetrahedron. The electrical scalar potentials at each of the four vertices within every tetrahedron, as well as at each of the six mid-edge nodes, are ascertained by the solver. The potential value for each element is subsequently determined through a quadratic equation.

**Figure 3:**
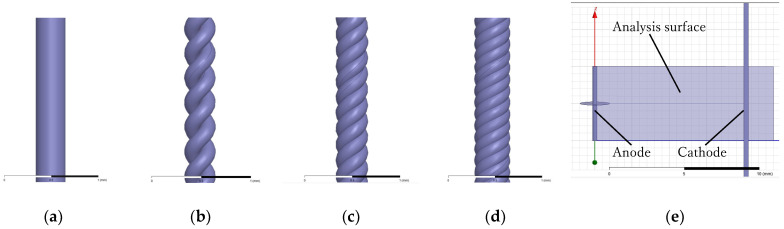
Model diagram depicting the anode configuration during the simulation of an electric field. The configurations include (**a**) a solid wire, (**b**) a 2-stranded wire, (**c**) a 3-stranded wire, and (**d**) a 4-stranded wire. (**e**) The arrangement of the anode and cathode, along with the analysis plane for the electric field. The separation between the electrodes measures 10 mm, and the analysis plane is positioned horizontally in relation to the anode. The reference bars at the base of the diagram denote scales of 1 mm and 10 mm for (**a**–**e**), respectively.

The development of three-dimensional models for the electrodes was accomplished through the application of Fusion360 (Autodesk, San Rafael, CA, USA). The anode constituted a rectilinear electrode, comprising either a solitary metallic wire or intricately interlaced helical strands. The plate electrode, operating as a cathode, exhibited dimensions measuring 15 mm in length, 15 mm in width, and a thickness of 0.5 mm. The simulation of the electric field distribution entailed subjecting the anode to a peak voltage of 20 kV. Analyses of electric field distributions were executed for various wire electrode configurations, encompassing a solitary wire and stranded wires. Figure 3 presents a diagrammatic representation of both a solid wire and stranded wires, showing the disposition of anodes and cathodes within the framework of the electric field simulation. The entwined wire electrodes featured individual wire diameters of 0.16 mm and a twisting rate of 166.7 T/m. To facilitate our analysis, as depicted in Figure 3d, we selected an analysis plane that intersected the center of the entire electrode, ensuring it was parallel or perpendicular to the wire electrode. This enabled the generation of electric field distribution diagrams for that specific plane. 

To conduct simulations in ANSYS Maxwell, it was imperative to define the region surrounding the object under investigation. In this study, the specified area for simulations was designated to be filled with air. By default, this simulator generates a mesh comprising 1000 elements; however, for the purposes of this study, the element count was augmented to 5000 to facilitate the creation of a smoother mesh and the generation of a more continuous vector field.

### 2.6. Statistical Analysis

In the pursuit of statistical scrutiny, a multiple regression analysis was conducted upon the designated dataset employing VBA within Microsoft Excel 2019. Specifically, the dependent variable was designated as any column within the Excel sheet, while multiple other columns were assigned as independent variables. The LINEST function, a method employing the least squares approach, was utilized for this analysis. It facilitated the computation of a straight line that optimally fits the specified data, yielding an array describing this line to ascertain a correction term. The *p*-value was derived from the t-Statistic, and the statistical significance of each independent variable was duly assessed. *p* values were computed at the 95% confidence level.

## 3. Results

### 3.1. Disinfection Rates under Different Voltage Waveforms

**Figure 4:**
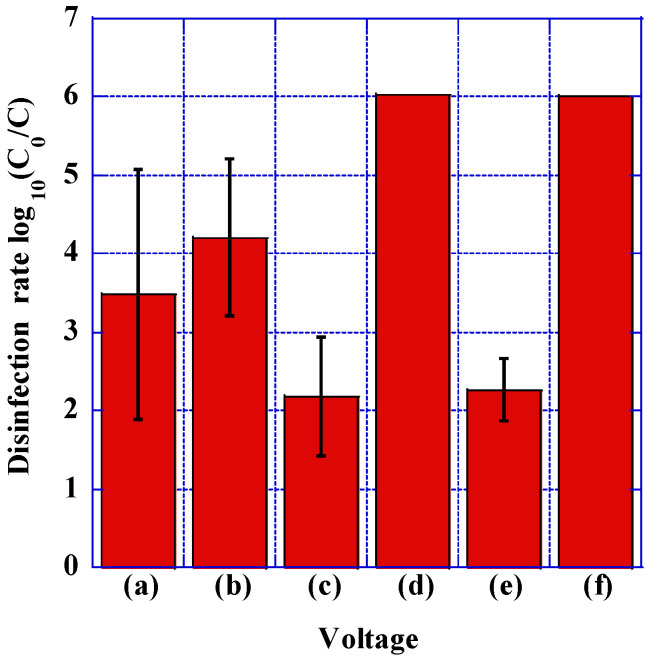
Disinfection rates upon applying AC, DC voltage, and pulse voltage for 20 min (*N* = 5). (**a**) DC 15 kV, (**b**) DC 20 kV, (**c**) Pulse 15 kV, (**d**) Pulse 20 kV, (**e**) AC 15 kV, (**f**) AC 20 kV. While at 15 kV application, DC voltage exhibited a higher disinfection rate compared with pulse voltage, the disinfection rate for pulse voltage significantly increased and surpassed DC voltage at 20 kV application. Error bars in the figures represent the standard deviation. The detection limit of viable counts is indicated without error bars.

We have identified disinfecting effects resulting from the application of DC, pulsed, and AC voltages. These findings are graphically represented in Figure 4. For instance, when subjecting the electrodes to a 15 kV DC application, the disinfection rate reached 3.48 logarithmic units, which notably exceeded the 2.18 logarithmic units achieved with pulsed 15 kV. Nevertheless, upon elevating the voltage to 20 kV, the disinfection rate of the DC voltage reached 4.21 logarithmic units, whereas the disinfection rate of the pulsed voltage surged to an impressive 6.01 logarithmic units, approaching the detection limit. This indicates that the change in disinfection rate with the increasing DC voltage was only 0.78, while the disinfection rate exhibited a noteworthy augmentation of 3.5 logarithmic units with the escalating pulse voltage.

When subjected to an AC or pulsed voltage at 15 kV, both the AC voltage and pulsed voltage demonstrated disinfection rates of 2.18 logarithmic units and 2.26 logarithmic units, respectively, without any discernible disparity between them. Conversely, at 20 kV, the disinfection rates escalated to 6.01 logarithmic units for AC voltage and a noteworthy 6.03 logarithmic units for the pulsed voltage. In both instances, the absence of viable bacteria was incontrovertible, effectively attaining the detection threshold. Furthermore, the initial bacterial concentrations of 10^3^ CFU/mL and 10^8^ CFU/mL demonstrated no change in the disinfection efficacy, almost the same as that observed at 10^6^ CFU/mL.

**Figure 5:**
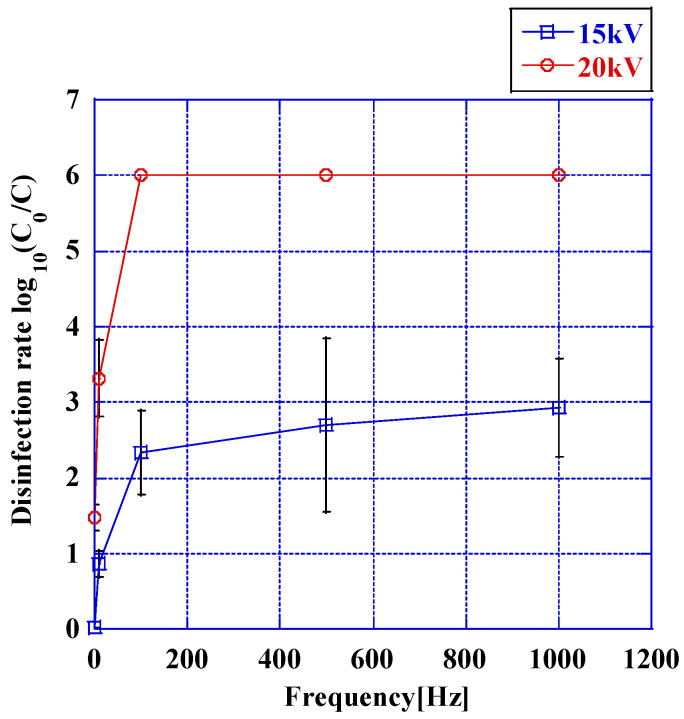
Disinfection rates upon the application of pulse voltages of 15 kV and 20 kV for 20 min at voltage frequencies of 1, 10, 100, 500, and 1 kHz (*N* = 5). The disinfection rate increased with the rise in frequency. Furthermore, at the same frequency, an increase in disinfection rate was observed with the elevation of applied voltage. Error bars in the figures represent the standard deviation. The detection limit for viable counts is presented without error bars.

The disinfection rate manifested a positive correlation with the frequency during the maintenance of a constant pulse voltage applied to the anode, as explicated in Figure 5. The aforementioned figure delineates the disinfection rates corresponding to pulse voltages of 15 kV and 20 kV across a range of frequencies spanning from 1 Hz to 1 kHz. At a voltage magnitude of 20 kV, a disinfection efficacy of 6.0 logarithmic units was achieved at frequencies exceeding 100 Hz. Conversely, at a voltage of 15 kV, the disinfection efficacy persisted at 3.92 logarithmic units even with an increase in frequency up to 1 kHz. The temperature of the bacterial suspension obtained from the anode exhibited minimal variation under both conditions, maintaining at 14.5 ± 1.3 °C before and after the imposition of voltage.

### 3.2. Disinfection Using Solid Wire and Stranded Wire Electrodes

**Figure 6:**
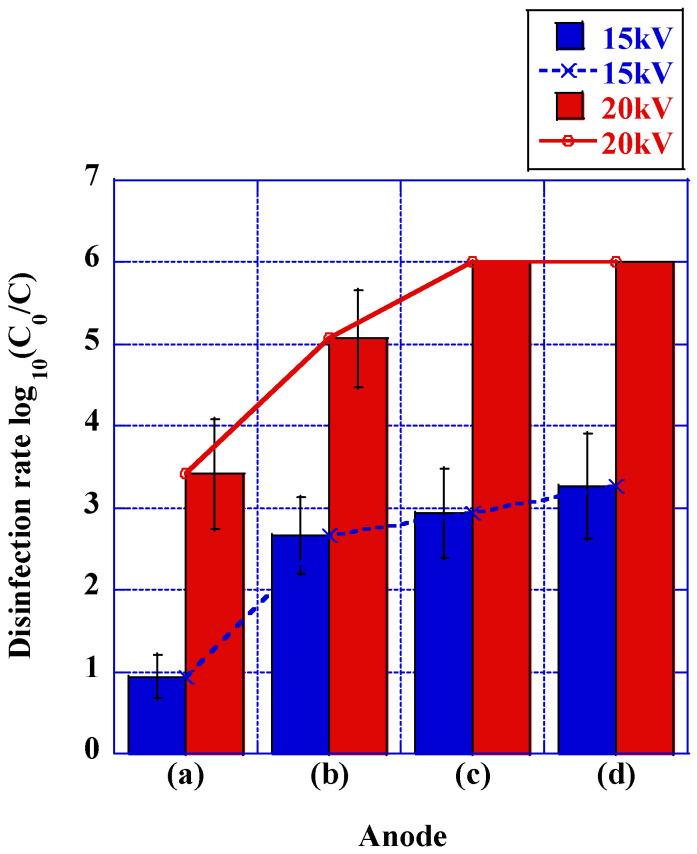
Comparison of disinfection rates between solid wire and stranded wire (*N* = 5). (**a**) a solid wire, (**b**) a 2-stranded wire, (**c**) a 3-stranded wire, and (**d**) a 4-stranded wire. An increase in disinfection rate was observed when transitioning from a solid wire to two stranded wires, as shown in Figure 6a,b. Error bars in the figures represent the standard deviation. The detection limit of viable counts is without error bars.

The modulation of disinfection rates concomitant with the augmentation of the quantity of stranded wires employed as anodes is delineated in Figure 6. At a voltage of 15 kV, the disinfection rate demonstrated a minimal value of 0.94 logarithmic units for solid wires but underwent a substantial increase to 2.66 logarithmic units for stranded wires. The disinfection rate experienced a simultaneous escalation with an augmentation in the quantity of stranded wires, culminating in a disinfection rate of 3.27 logarithmic units in the scenario of four stranded wires. At a voltage of 20 kV, the disinfection rate for stranded wires showed a more significant increase compared with solid wires, aligning with the trend observed at 15 kV. Upon augmenting the number of strands, the disinfection rate achieved 6.00 logarithmic units, corresponding to the detection limit, for a configuration of three strands.

### 3.3. Electric Field Analysis of Solid Wire and Stranded Wire Electrodes

**Figure 7:**
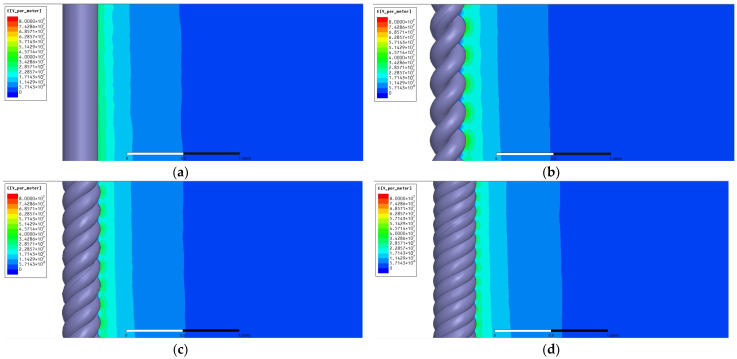
Electric field distribution arising from either a singular wire or a helically twisted wire configuration. The graphical representation showcases the electric field distribution pertaining to (**a**) a solid wire, (**b**) a 2-stranded wire, (**c**) a 3-stranded wire, and (**d**) a 4-stranded wire.

Figure 7 illustrates the outcomes of the electric field distribution simulation concerning the anode, comparing conditions where the anode assumed the form of either a solid wire or a stranded wire. The electric field distribution of the solid wire demonstrated uniformity in the proximity of the anode, gradually diminishing towards the cathode. Conversely, the electric field distribution of the two-strand wire exhibited non-uniformity, contingent upon the configuration of the anode. Specifically, it was corroborated that the electric field concentrated on the convex surface of the electrode. The electric field distribution gradually attenuated towards the cathode. As the number of strands increased, it was observed that the electric field concentrated on the convex portion of the electrode shape but gradually diminished, with no discernible disparity in the reduction-to-electrode-distance ratio.

**Figure 8:**
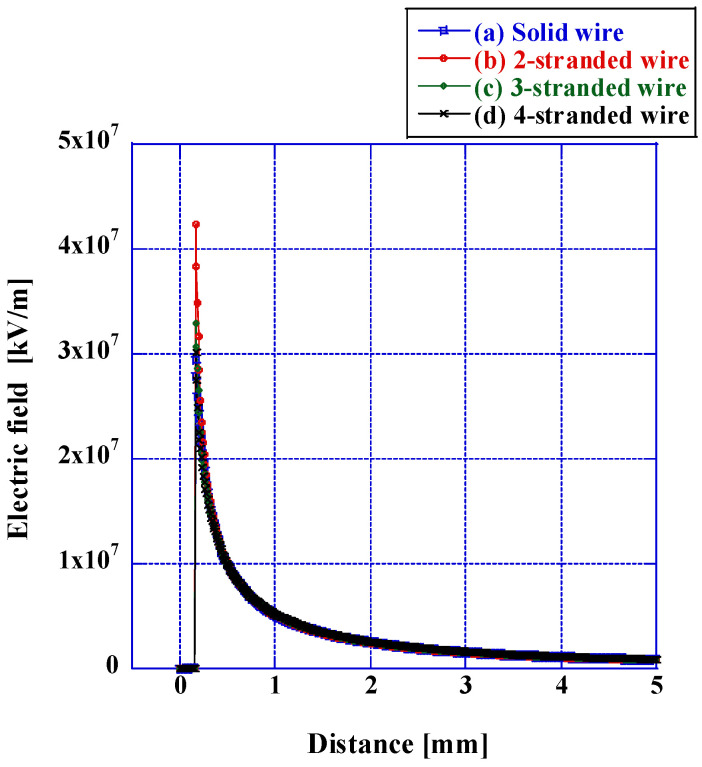
The variation in the maximum electric field under the conditions of a solid wire and a twisted wire anode configuration. The electric field profiles are delineated for (a) a solid wire, (b) a 2-stranded wire, (c) a 3-stranded wire, and (d) a 4-stranded wire. The electric field reached its maximum potency at the surface of the anode and exhibited a subsequent decline towards the cathode. The electric field intensity was greater for stranded wires compared with solid wires, yet it diminished with an escalation in the number of stranded wires.

Figure 8 portrays the variation in the maximum electric field from the anode’s periphery to the cathode. The electric field on the electrode surface measured 2.94 × 10^7^ kV/m for a solid wire and 4.23 × 10^7^ kV/m for two stranded wires, reflecting an augmentation exceeding 1.4 times. When the number of intertwined wires escalated to three and four, the field strengths measured 3.29 × 10^7^ kV/m and 3.01 × 10^7^ kV/m, respectively. The maximum field strength diminished with the increasing number of strands. Nevertheless, the extent of decrease in the field strength remained nearly constant irrespective of the anode geometry. At a distance of 1 mm from the field surface, the value was approximately 5.1 × 10^7^ kV and remained essentially unaltered. In summary, the simulation findings indicate that the electric field potency proximate to the electrode escalates upon transitioning the anode from a solid wire to a stranded wire configuration. Conversely, the electric field strength attenuates with an increase in the number of stranded wires. The electric field vigor diminishes from the anode to the cathode, with the extent of reduction remaining relatively uniform irrespective of the anode’s morphological configuration. 

The distribution of the electric field from the electrode surface to the cathode was examined. The electric field intensity in proximity to the anode exhibited uniformity in the case of a solid wire, whereas the electric field became concentrated on the convex surface of the stranded wire electrode. The electric field, spanning from the anode to the cathode, exhibited a gradual reduction over distance. The scale in the illustration is denoted by the bar, measuring 1 mm.

## 4. Discussion

The application of pulse voltage generates Maxwell stress in the cell membrane, resulting in pore formation, the release of intracellular contents, and, ultimately, cell death [44,48]. Miyazaki et al. discussed the creation of pores in cell membranes influenced by an external electric field and proposed the hypothesis that electrical energy entering the pore increases the temperature within the pore, causing the lipid membrane to separate [44]. Their findings suggest that the electric potential gradient across the cell membrane leads to a rapid increase in pore temperature due to electric current flow. This creates pressure that disrupts the phospholipids and membrane structure. However, under a constant external electric field, power density decreases as pores expand, eventually reaching a saturation point. In our current experiment, the increase in the disinfection rate with an increasing DC voltage is less pronounced than the increase in the disinfection rate with an increasing pulse and an AC voltage from 15 kV to 20 kV. This phenomenon likely results from pore expansion saturation under the electrostatic field when DC voltage is applied. 

Schoenbach et al. reported that bipolar pulses were more effective for inactivation than monopolar pulses [43], while Beveridge et al. demonstrated the superiority of monopolar pulses over bipolar pulses [49]. However, the results of our current experiment showed no significant difference in the disinfection effect between the AC voltage and the positive pulse voltage. Nevertheless, our findings confirm that the increase in the disinfection rate aligns with previous studies, occurring due to the rise in electric field intensity applied across all voltage types, including DC, AC, and pulse voltages [34,35,36,40,41,42,43,44,45,50,51,52,53,54,55,56]. We also observed a correlation between the bacterial disinfection rate and the applied voltage frequency. The increase in frequency likely led to more frequent interactions between the electric field and the cell membrane, resulting in a higher disinfection rate. Furthermore, we noted an increase in the disinfection rate with higher field strength at the same frequency.

Multiple regression analysis was employed to discern correlations between the disinfection efficacy of *E. coli* and the corresponding electrical parameters, namely initial voltage, frequency, and the quantity of anode strands. The implementation of multiple regression analysis facilitated a comprehensive elucidation of the relationship between the reduction rate of *E. coli* and each electrical parameter, including initial voltage, frequency, and the number of anode strands, as detailed in Table 1. The adjusted coefficient of determination (R^2^ value) for *E. coli* reduction (0.62) was not weak; the coefficients linked to *E. coli* reduction exhibited statistically significant values (*p* < 0.05), signifying the substantial impact of these parameters on the reduction rate. Conversely, augmenting the number of anode strands in the disinfection process, devoid of voltage application, failed to yield a reduction in the number of *E. coli*. Consequently, it was conjectured that the initial voltage played a pivotal role in their inactivation (*p* < 0.05). Furthermore, frequency demonstrated significance (*p* < 0.05) concerning the diminished quantity of *E. coli*. Additionally, the correlation between the number of anode strands and the reduction in *E. coli* numbers was more pronounced compared with the frequency.

The simulation outcomes pertaining to electric field strength indicate an augmentation in the field strength of the stranded wire in comparison with that of the solid wire. This phenomenon can be ascribed to the configuration of the electrode surface. To elucidate, the irregularities present on the electrode surface expedite the focalization of the electric field and are thereby postulated to enhance the intensity of the electric field. The results obtained from the electric field simulation of stranded conductors unveil that the electric field intensity proximate to the anode registers values of 4.23 × 10^7^ V/m, 3.29 × 10^7^ V/m, and 3.07 × 10^7^ V/m, exhibiting a reduction concomitant with the increase in the number of stranded wires. As discernible from the shape depiction of the stranded wire in Figure 3, the larger the separation between the concave and convex segments, the facilitation of electric charge accumulation in the convex segment becomes apparent, thereby resulting in a heightened charge density and, subsequently, an augmented electric field strength. It is postulated that with an increase in the number of stranded wires, the topography of the electrodes tends towards planarity, resulting in a concomitant reduction in field strength. As previously stated, while the prevailing understanding posits a positive correlation between disinfection rate and electric field intensity, the disinfection rate exhibited negligible variation upon the augmentation of stranded wire quantities. Kajiwara et al. and Chen et al. delineated that the application of a pulsed voltage measuring 50 kV/cm resulted in the inactivation of approximately 5 to 6 logarithmic units vis-à-vis *Enterobacter Aerogenes* (*E. Aerogenes*) and *E. coli* [50,57,58]. In this simulation, the electromagnetic field intensity remained consistently above 10^2^ kV/cm for all configurations of the stranded conductors. This magnitude proves adequate for the incapacitation of Gram-negative bacteria, leading to the presumption that there exists no discernible distinction in disinfection potency contingent upon the number of stranded wires. The geometric configuration of the stranded wire is deemed consequential in augmenting the disinfection efficacy of the wire. Stranded wires engender uneven topographies on the electrode surface when juxtaposed with solid wires. These irregularities induce heightened turbulence in airflow and increased intricacy in wind direction, thereby mitigating the overall flow velocity [59,60]. The resultant reduction in flow prompts a more frequent application of the electric field per unit of time, posited to correlate with an escalation in disinfection intensity. Prior investigations have evidenced that a decline in wind velocity correlates positively with an augmentation in disinfection intensity [61]. Consequently, although an increase in the number of strands results in a slight reduction in field strength, the overall impact does not cause a decrease in the disinfection rate. The turbulentization of airflow proximate to the electrodes diminishes air velocity, culminating in a marked escalation in disinfection intensity. The disinfection rate of a stranded wire at a voltage of 20 kV achieves a logarithmic value of 6.0 at a frequency of 10 Hz, surpassing that at a frequency of 100 Hz when the electrode is a solid wire. Henceforth, the energy efficiency of the stranded wire for disinfection is exceptionally high.

The Decimal Reduction Value (D-value) is a crucial parameter in assessing the effectiveness of various sterilization methods. For instance, Nomura et al. demonstrated a D-value of 0.77 min for *Geobacillus stearothermophilus* (*G. stearothermophilus*) in sterilization mode using low-temperature ozone/hydrogen peroxide-mixed gas exposure [62]. Similarly, Cardoso et al. highlighted the efficiency of hydrogen peroxide in achieving a 7 decimal reduction when the contact time was extended to 16 s [63]. Moreover, the D-value varies depending on the sterilization method and the target microorganism, as noted by Luechapattanaporn et al. [64]. For instance, Murphy et al. (2004) reported D-values of *E. coli* O157:H7 at 55 to 70 °C, ranging from 33.44 to 0.048 min, demonstrating the impact of temperature on the D-value [65]. Additionally, Wang et al. indicated a D-value of <0.72 min for *E. coli* O157 when exposed to acidic electrolyzed water for 5 min, highlighting the effectiveness of this method in reducing *E. coli* populations [66]. 

Prior research findings have demonstrated that, by employing a 20 kV pulsed voltage over 10 min with a solid-wire anode, a disinfection rate exceeding 6.0 log was attained [61]. In the current study, utilizing a stranded wire anode, the disinfection rate was nearly twice that achieved with a solid-wire anode, with the calculated D-value approximating 0.94 min. This D-value is of a similar order of magnitude as those observed in the context of gaseous and acidic electrolyzed water, suggesting the viability of pulse voltage-based disinfection technology as a potential approach for space disinfection.

The pulsed electric field spatial disinfection techniques explored in this study were compared with alternative methods to assess their efficacy. Diverse strategies for spatial sterilization have undergone manifold academic investigations. For instance, Zhao et al. introduced an intelligent sterilization robotic system employing chlorine dioxide for aerosolized disinfection, thereby exemplifying the potential of sophisticated technological interventions in this domain [67]. Additionally, Liu et al. deliberated upon the utilization of thermally sprayed photocatalytic coatings for biocidal objectives, accentuating the deployment of advanced coatings in disinfection across diverse spatial environments [68]. Furthermore, Feng et al. executed numerical simulations assessing the performance of an electrostatic disinfector (ESD) in airborne disinfection within indoor settings, signifying the promise inherent in electrostatic discharge-based techniques for spatial disinfection [69]. Collectively, these studies yield insights into an array of strategies for spatial disinfection, encompassing the utilization of intelligent sterilization systems, cutting-edge coatings, and electrostatic-based disinfection approaches. The proposition articulated in this experiment, advocating for space disinfection through the application of an electric field, emerges as innovative and sets itself apart as a pioneering technique, thereby endowing the findings of this paper with considerable novelty. Furthermore, the experimental findings demonstrated the attainment of a disinfection efficacy reaching 6.0 logarithmic units, indicative of the potential realization of a Sterility Assurance Level (SAL) of 10^−6^ through the utilization of this technology. The methodologies and International Organization for Standardization (ISO) standards required to assess aerosol sterilization cover a range of considerations, including procedure validation, scrutiny of filtration efficacy, safety assessment, and microbial exposure testing [70,71,72]. These prescribed standards and methodologies are indispensable in ensuring both the efficacy and safety of the aforementioned sterilization process.

## 5. Conclusions

- The objective of this study was to achieve aerosol disinfection through the application of a pulsed voltage and to scrutinize the distribution of the electric field across diverse electrode configurations. Consequently, the ensuing principal discoveries were ascertained:
- Disinfection rates exhibited an augmentation concomitant with the escalation of the applied voltage.
- When the voltage was elevated from 15 kV to 20 kV, a discernible variance manifested in the augmentation of disinfection rates between pulsed and DC voltages. Nonetheless, no significant distinction in the disinfecting efficacy was observed between the pulsed and AC voltages.
- The disinfecting efficacy witnessed an amplification in tandem with an escalation in the frequency of the pulsed voltage.
- Altering the electrode geometry from a solid wire to two stranded wires resulted in an increase in the disinfection rate. The augmentation in disinfection rate with stranded wires was substantial, surpassing 2.0 logarithmic units in comparison with the solid wire electrode.
- Electric field analysis divulged a marginal reduction in field strength with an augmentation in the number of stranded wires. Nevertheless, the actual disinfection potency exhibited an escalation. This phenomenon may be attributed to an upsurge in the frequency of electric field applications per unit of time, attributable to diminished air velocity induced by the electrode configuration.

## Figures and Tables

**Table 1:** Multiple regression analysis between each reduction ratio of *E. coli* count and each electrical parameter. The adjusted coefficient of determination (R^2^ value) for *E. coli* reduction was 0.62.

	Coefficient	t Stat	*p*-Value
**Intercept**	5.20 × 10^6^	22.9	3.57 × 10^−59^
**Number of strands**	−3.47 × 10^5^	−2.6	8.91 × 10^−3^
**Voltage**	−2.34 × 10^5^	−11.7	1.33 × 10^−24^
**Frequency**	−9.63 × 10^2^	−2.2	2.76 × 10^−2^

## Data Availability

Data are contained within the article.

## References

[1] Vella F., Senia P., Ceccarelli M., Vitale E., Maltezou H., Taibi R., Ledda C. (2020). Transmission mode associated with coronavirus disease 2019: A review. Eur. Rev. Med. Pharmacol. Sci..

[2] Myojo T., Okada T. (2021). Aerosol Transmission of COVID-19 and Effectiveness of Masks. J. Aerosol Res..

[3] Takekagawa N. (2021). Aerosol, Droplet Transmission, and Airborne Transmission. J. Aerosol Res..

[4] Kousaka Y., Nomura T., Naito M. (2020). The Possibility of the Aerosol Infection of Corona Disease COVID-19—Analysis from the Viewpoint of Particle Technology. J. Soc. Powder..

[5] Yanagi U. (2018). Design of air-conditioning and ventilation system in hospital-acquired infection prevention. Jpn. J. Clin. Ecol..

[6] Takada K., Furukawa T., Ueno T. (2023). Study on efficiency of aerosol disinfection by PEF. 1st Kosen Res. Int. Symp..

[7] Yamaguchi M., Isawa K., Yamada Y., Kawakami R., Tomioka K. (2015). Examination of the Anti-Microbe Measure with the Chlorine-Based Chemicals: Evaluation of Sterlizing Efficacy of Ultrasonic Fogging with Hypochlorite or Slightly Acid Electrolzed Solutions in an Actual Large Space. J. Environ. Eng. (Trans. AIJ).

[8] Tatsumi T., Ikeguti A., Sasaki K., Oginol A., Nishi Y., Goto M. (2010). Effect of Fogging with Solution of Didecyl Dimethyl Ammonium Chloride (DDAC) on Aerial Bacteria and Dust in Floor Feeding Meat-type Chicken Windowless House Ventilated Cross Wise under Negative Pressure. J. Jpn. Soc. Poult. Dis..

[9] Tatsumi T., Sasaki K. (2010). Effect of Spraying with a Solution of Didecyl Dimethyl Ammonium Chloride (DDAC) on Aerial Bacteria in Layer Cage-Feeding Windowless House Cross-Ventilated Under Negative Pressure. J. Jpn. Soc. Poult. Dis..

[10] Imanishi Y., Furuta K. (1991). Factors Affected for Efficacy of Disinfectant Solutions Sprayed like a Mist. Jpn. Poult. Sci..

[11] Naito S. (1995). Inhibition of Food Microorganisms by Application of Ozone and UV. Jpn. J. Food Microbiol..

[12] Ikeda A., Kawai Y., Esaki K., Nakayama S. (1998). Sterilization of Vegetables Preserved at Low Temperature with Low Ozone Concentration. J. Soc. High. Technol. Agric..

[13] Kusumoto N., Watanabe A., Hasunuma Y., Hiraoka S., Kawashima N., Tokuoka Y. (2020). Bactericidal activity against Bacillus atrophaeus by ultraviolet-ozone generation method using microwave plasma. Mater. Technol..

[14] Seki H. (2015). The Inactivation of Virus and Microbe in the Air & CT Value. Urban. Pest. Manag..

[15] Sung M., Kato S., Yanagi U. (2009). Evaluation of surface and air disinfection effects using UV radiation simulation. J. Environ. Eng..

[16] Watanabe Y., Nawa H., Koike N. (1997). The Follow-up Study on Effects of Ultraviolet Air Disinfection System against Airborne Bacteria. Jpn. J. Environ. Infect..

[17] Nishikiori K. (1997). The Utilization of Ultraviolet Ray and Electron Beam. J. Illum. Eng. Inst. Jpn..

[18] Sasaki Y., Funayama H. (2010). UV Sterilization Characteristics of E coli and Bacillus Subtilis Spores. Res. Rep. Natl. Inst. Technol. Akita Coll..

[19] Takeuchi Y. (1995). Deodorization technology using ozone lamp. Jpn. J. Swine Sci..

[20] Yamabe C. (2006). Ozone Generation Technologies and Their Applications. The transactions of the institute of Electrical Engineers of Japan. Fundam. Mater. Soc..

[21] Ohkubo T., Kanazawa S., Nomoto Y., Chang J.S. (1991). The Effect of CO2 Concentration in Air on Ozone Generation by Corona Discharges in a Wire-Plate Electrode System. J. Inst. Electrost. Jpn..

[22] Uchino T. (2013). Physical Disinfection Technique for Agricultural Products. J. Jpn. Soc. Agric. Mach..

[23] Nakamura H., Furuhashi M. (1986). Ultraviolet Irradiation of Air Sterilization under Dynamic Airflow System: Bacterial Aerosols Behavior and Inactivation. J. Aerosol. Res..

[24] Kato S., Sung M., Yanagi U., Takagi T., Yanagihara R., Ida H., Tanaka T., Asai M. (2008). Application of UVGI system to Indoor Air in North America. Soc. Heat. Air-Cond. Sanit. Eng. Jpn..

[25] Oguma K., Koshio M., Lohwacharin J., Takizawa S. (2017). Effects of Suspended Particles in Water on Efficiency of UV Disinfection. J. Jpn. Soc. Water Environ..

[26] Mizuno T. (2011). Ozone is such a substance (Chemistry of Ozone). Chem. Educ..

[27] Akaike T. (1989). Surface Chemistry of Bioelectronic Materials—Approach to Artificial Mitochondria and Highly Functional Artifical Organ Devices. J. Surf. Sci. Soc. Jpn..

[28] Shoji S. (2003). Micro/Nanosystems for Chemical/Biochemical Applications—Applications of MEMS and Top-down Nanotechnologies for Chemistry and Biotechnology. Hyomen Kagaku.

[29] Kinoshita A., Omukai T., Komada F., Utsumi Y. (2010). High density cell culture using micro 3D structure. J. Jpn. Inst. Electron. Packag..

[30] Onoda M., Abe Y., Tada K., Kawakita Y., Fujisato T., Uto S. (2012). Conductive Polymers as Bioelectronic Materials. IEEJ Trans. EIS.

[31] Suzuki T. (2010). A Cell Array Fabricated by Assembly-Free Multidirectional Photolithography. J. Jpn. Inst. Electron. Packag..

[32] Sale A.J.H., Hamilton W.A. (1967). Effects of high electric fields on microorganisms: I. Killing of bacteria and yeasts. Biochim. Biophys. Acta (BBA)—Gen. Subj..

[33] Sato T., Murakami Y., Muramoto Y. (2019). Effect of D.C. High Electric Field Application on Sterilization of Escherichia coli in Ice. Trans. Jpn. Soc. Refrig. Air Cond. Eng..

[34] Ishida N., Ohshima T., Sato M. (2004). Characteristics of Pulsed Electric Field Inactivation System Using Spiral Electrode Configuration. Jpn. J. Food Eng..

[35] Uemura K., Inoue T., Hoshino T. (2016). Inactivation of Microorganisms in Liquid Foods by High Electric Field Alternating Current. Nippon. Shokuhin Kagaku Kogaku Kaishi.

[36] Nakamura G., Oishi R., Nuki H., Katsuki S., Masuda N., Shimizu Y. (2021). Influence of Fat Globules on Pulsed Electric Field Sterilization of Milk. Trans. Inst. Electr. Eng. Jpn. A.

[37] Lee K., Paek K.H., Ju W.T., Lee Y. (2006). Sterilization of bacteria, yeast, and bacterial endospores by atmospheric-pressure cold plasma using helium and oxygen. J. Microbiol..

[38] Nakamura M., Nishikawa K. (2009). Principle and Application of Sanitization Function of Plasma Cluster Ions. SEIKATSU EISEI (J. Urban Living Health Assoc.).

[39] Zhang L., Guo Y., Chang X., Yao Z., Wei X., Feng Z., Zhang D., Zhou Q., Wang X., Luo H. (2022). In-duct grating-like dielectric barrier discharge system for air disinfection. J. Hazard. Mater..

[40] Hayamizu A., Tenma T., Mizuno A. (1989). Destruction of Yeast Cells by Pulsed High Voltage Application. Int. J. Plasma Environ. Sci. Technol..

[41] Ramaswamy R., Prabu Ramachandran R., Gowrisree V. (2019). High Voltage Pulsed Electric Field Application Using Titanium Electrodes for Bacterial Inactivation in Unpurified Water. Jpn. J. Food Eng..

[42] Kitajima N., Akuzawa E., Ueda K., Ohshima T., Sato M. (2007). Inactivation System Using Textile Electrode by Pulsed Electric Field. Jpn. J. Food Eng..

[43] Schoenbach K.H., Joshi R.P., Stark R.H., Dobbs F.C., Beebe S.J. (2000). Bacterial Decontamination of Liquids with Pulsed Electric Fields. IEEE Trans. Dielect. Electr. Insul..

[44] Miyazaki D., Toyomitsu H., Katano K., Kazue S., Katsuki S. (2020). Experimental and Aanalytical Considerations of Pulse Waveform for Efficient Sterilization Using Pulsed Electric Field. Proc. Inst. Electrost. Jpn..

[45] Bai-Lin Q., Qinghua Z., Barbosa-Canovas G.V., Swanson B.G., Pedrow P.D. (1994). Inactivation of microorganisms by pulsed electric fields of different voltage waveforms. IEEE Trans. Dielectr. Electr. Insul..

[46] Zukeran A., Sawano H., Ito K., Oi R., Kobayashi I., Wada R., Sawai J. (2018). Investigation of inactivation process for microorganism collected in an electrostatic precipitator. J. Electrost..

[47] Kakutani K., Matsuda Y., Nonomura T., Takikawa Y., Takami T., Toyoda H. (2021). A Simple Electrostatic Precipitator for Trapping Virus Particles Spread via Droplet Transmission. Int. J. Environ. Res. Public Health.

[48] Katsuki S. (2003). Bacteria Decontamination by Pulsed Power. J. Plasma Fusion. Res..

[49] Beveridge J.R., Wall K., MacGregor S.J., Anderson J.G. The Influence of Pulse Duration of Monopolar and Bipolar Profile Pulsed Electric Fields on the Inactivation of Obesumba Cterium Rroteus—A Spoilage Microorganism. Proceedings of the Conference Record of the Twenty-Sixth International Power Modulator Symposium, 2004 and 2004 High-Voltage Workshop.

[50] Kajiwara T., Oide T., Baba K., Ohnishi N., Katsuki S., Akiyama H., Sasahara R., Inoue K. (2015). Inactivation of Enterobacter Aerogenes in Carboxymethyl Cellulose Solution Using Intense Pulsed Electric Fields (IPEF) Combined with Moderate Thermal Treatment. IEEE Trans. Dielect. Electr. Insul..

[51] Gusbeth C., Frey W., Volkmann H., Schwartz T., Bluhm H. (2009). Pulsed electric field treatment for bacteria reduction and its impact on hospital wastewater. Chemosphere.

[52] Baba K., Kajiwara T., Watanabe S., Katsuki S., Sasahara R., Inoue K. (2018). Low-Temperature Pasteurization of Liquid Whole Egg Using Intense Pulsed Electric Fields. Electron. Comm. Jpn..

[53] Ricci A., Parpinello G.P., Versari A. (2018). Recent Advances and Applications of Pulsed Electric Fields (PEF) to Improve Polyphenol Extraction and Color Release during Red Winemaking. Beverages.

[54] Sakurauch Y., Kondo E. (1980). Lethal Effect of High Electric Fields on Microorganisms. J. Agric. Chem. Soc. Jpn..

[55] Saito K., Nozawa Y., Minamitani Y., Fukata H. (2017). Development of Non-Thermal Sterilization Treatment System without Impacting to Enzyme in Vegetable Drink by Pulsed Electric Field. IEEJ Trans. FM.

[56] Ishida N.M., Sugiarto A.T., Ohshima T., Sato M. (2003). Killing Effect of Concentrated Pulsed Electric Field Apparatus. Jpn. J. Food Eng..

[57] Kajiwara T., Oide T., Katsuki S., Sakugawa T., Akiyama H. Liquid sterilization using intense electrical pulses combined with thermal post-treatment. Proceedings of the 2014 IEEE International Power Modulator and High Voltage Conference (IPMHVC).

[58] Chen J., Tao X., Sun A., Wang Y., Liao X., Li L., Shi Z. (2014). Influence of pulsed electric field and thermal treatments on the quality of blueberry juice. Int. J. Food Prop..

[59] Kubokawa H., Imoto S., Ogi I., Kitashima T., Tanabe K. (2004). Countermeasures for Wind Noise from Overhead Transmission Line. Wind. Eng..

[60] Shimojima K. (1994). Aerodynamic Noise generated by Overhead Transmission Line; Its Prediction and Control. J. INCE Jpn..

[61] Ueno T., Takada K., Furukawa T., Sakugawa T. Aerosol Sterilization by Impulse High Voltage with Different Electrode Structures. Proceedings of the 2022 International Conference on Electrical, Computer and Energy Technologies (ICECET).

[62] Nomura Y., Yamamura J., Fukui C., Fujimaki H., Sakamoto K., Matsuo K., Kuromatsu H., Kikuchi Y., Haishima Y. (2021). Performance Evaluation of Bactericidal Effect and Endotoxin Inactivation by Low-temperature Ozone/Hydrogen Peroxide Mixed Gas Exposure. J. Biomed. Mater. Res. Part B Appl. Biomater..

[63] Cardoso C.F., Faria J.d.A.F., Walter E.H.M. (2011). Modeling of Sporicidal Effect of Hydrogen Peroxide in the Sterilization of Low Density Polyethylene Film Inoculated With Bacillus Subtilis Spores. Food Control.

[64] Luechapattanaporn K., Wang Y., Wang J., Tang J., Hallberg L.M., Dunne C.P. (2006). Sterilization of Scrambled Eggs in Military Polymeric Trays by Radio Frequency Energy. J. Food Sci..

[65] Murphy R.Y., Beard B.L., Martin E.M., Duncan L.K., Marcy J.A. (2004). Comparative Study of Thermal Inactivation of *Escherichia Coli* O157:H7, *Salmonella*, and *Listeria Monocytogenes* in Ground Pork. J. Food Sci..

[66] Wang H., Feng H., Luo Y. (2006). Dual-Phasic Inactivation of *Escherichia coli* O157:H7 with Peroxyacetic Acid, Acidic Electrolyzed Water and Chlorine on Cantaloupes and Fresh-cut Apples. J. Food Saf..

[67] Zhao Y., Huang H., Chen T., Chiang P., Chen Y., Yeh J., Weng W. (2021). A smart sterilization robot system with chlorine dioxide for spray disinfection. IEEE Sens. J..

[68] Liu Y., Huang J., Feng X., Li H. (2020). Thermal-sprayed photocatalytic coatings for biocidal applications: A review. J. Therm. Spray Technol..

[69] Feng Z., Cao S., Wang J., Kumar P., Haghighat F. (2021). Indoor airborne disinfection with electrostatic disinfector (esd): Numerical simulations of esd performance and reduction of computing time. Build. Environ..

[70] (2013). Sterilization of Health Care Products—Radiation—Part 2: Establishing the Sterilization Dose.

[71] (2017). Basic Human Body Measurements for Technological Design—Part 1: Body Measurement Definitions and Landmarks.

[72] (2019). Fine Ceramics (Advanced Ceramics, Advanced Technical Ceramics)—Test Method for Air-Purification Performance of Semiconducting Photocatalytic Materials—Part 2.

